# Oral and Hand Hygiene Behaviour and Risk Factors among In-School Adolescents in Four Southeast Asian Countries

**DOI:** 10.3390/ijerph110302780

**Published:** 2014-03-07

**Authors:** Karl Peltzer, Supa Pengpid

**Affiliations:** 1 ASEAN Institute for Health Development, Madidol University, Salaya, Phutthamonthon, Nakhonpathom 73170, Thailand; E-Mail: supaprom@yahoo.com; 2 Department of Psychology, University of Limpopo, Turfloop, Sovenga 0727, South Africa; 3 HIV, AIDS, TB, and STIs (HAST), Human Sciences Research Council (HSRC), Pretoria 0001, South Africa

**Keywords:** tooth brushing, hand washing, risk factors, adolescents, Southeast Asian countries

## Abstract

The aim of this study was to investigate oral and hand hygiene behaviour and risk factors among 13 to 15 year-old in-school adolescents in four Southeast Asian countries. Data were collected by self-reported questionnaire from nationally representative samples (total 13,824) of school children aged 13 to 15 years in India, Indonesia, Myanmar and Thailand. Results indicate that overall, 22.4% of school children reported sub-optimal oral hygiene (<twice a day tooth brushing), 45.2% did not always wash their hands before meals, 26.5% after toileting and 59.8% washing their hands with soap (59.8%). In multivariate analysis, male gender, health risk behaviours and lack of protective factors were associated with sub-optimal tooth brushing, and lower socioeconomic status, health risk behaviours, psychological distress and lack of protective factors were found to be associated with sub-optimal hand washing hygiene behaviour. As a conclusion, the cross-national data on oral and hand hygiene behaviour from four Southeast Asian countries found sub-optimal hygiene behaviour. Several determinants of sub-optimal hygiene behaviour were identified that can inform programmes in order to improve oral and hand hygiene behaviour of this adolescent population.

## 1. Introduction

Oral hygiene (tooth brushing of at least twice a day) is one of the most important methods for the control and prevention of dental caries and periodontal diseases [1,2]. In some Southeast Asian countries a high prevalence of dental caries has been observed among adolescent school children, ranging from 27%–45.5% in India [2,3,4], 50% in Indonesia [5] and 70% in Thailand [6]. Oral hygiene (tooth brushing) varied from school children in India (25% to 61.9% cleaned their teeth two or more times a day) [3,7,8] to Thailand (tooth brushing at least once a day was claimed by 88%) [6]. The recommended tooth brushing (at least twice daily) prevalence among school children in studies in other regions found in nine African countries 77.3% [9] and in the Pacific in Vanuatu 62%, in Tonga 70% and Pohnpei 78% [10]. Sociodemographic factors (younger age [8], being male [9,11,12], low socioeconomic status [8,10,11,13,14,15,16], rural residence [7], health risk behaviours including smoking [16,17], alcohol and cannabis use [12], inadequate exercise [16], infrequent fruits and/or vegetables consumption [9,18], lack of protective factors [9,19,20], and psychological distress [9] have been identified as risk factors for poor oral hygiene among adolescents. In a study in India a significant association between overweight and obese children and caries prevalence was found [4].

Hand washing with soap alone averts 0.5–1.4 million deaths per year [21], yet, hand washing levels seems to be low among school-aged children. In rural India the prevalence of good hand-washing (defined as “washing hands with soap and water after defecation and before eating food”) was 32.1% [22], and in two schools of Bangalore and Kolkata, India, 86% reported that they washed hands before eating lunch [23]. Studies among school children in other regions found in nine African countries that 37.8% did not always wash their hands before eating, 41.6% did not wash hands after toilet or latrine use and 65% did not always wash hands with soap [9], and in some Pacific islands hand washing before eating ranged from 65%–70% [10]. Poor hand washing behaviour among school-aged children has been found to be associated with being male [10], lower socioeconomic status [10,22], health risk behaviours (substance use [10] and infrequent fruits and/or vegetables consumption [9]), psychological distress [10], and lack of protective factors [9,10]. Dorri *et al*. [24] found a valid theoretical model of the factors influencing general and oral hygiene behaviours in adolescents, and that a positive relationship was shown between oral and general hygiene behaviours among adolescents in Iran [25]. This finding may indicate that such a relationship may also exist in other communities, such as in Southeast Asia.

There is a lack of information on the prevalence of and relationship between oral and hand hygiene, other health risk behaviours and psychosocial factors among adolescent populations in Southeast Asian countries. Therefore, the aim of this study was to investigate oral and hand hygiene behaviour and risk factors among 13 to 15 year-old in-school adolescents in four Southeast Asian countries.

## 2. Methodology

### 2.1. Sample and Procedure

This paper involved secondary analysis of data from the Global School-Based Health Survey (GSHS) from four Southeast Asian countries (India, Indonesia, Myanmar and Thailand). All Southeast Asian countries from which GSHS datasets (with the module on oral and hand hygiene) were publicly available were included in the analysis. Details and data of the GSHS can be located at the Centers for Disease Control and Prevention [26]. The purpose of the GSHS is to collect data with self-completed questionnaires focusing on students from ages 13 to 15 years. A two-stage cluster sample design is used to collect data to represent all students from Grades 6 to 10 in the study country. At the first stage of sampling, schools were selected with probability proportional to their reported enrollment size, and in the second stage, classes in the selected schools were randomly selected (using a random start) and all students in selected classes are eligible to participate [26]. 

Students completed the self-administered questionnaire during one classroom period under the supervision of trained survey administrators and recorded their responses to each question on an answer sheet suitable for computerized scanning [26].

Social and economic characteristics of the four participating Southeast Asian study countries are shown in Table 1. Two countries were lower middle income (India and Myanmar) and two countries (Indonesia and Thailand) upper middle income countries. Indonesia and Thailand also had higher mean years of schooling, lower proportions of children with underweight and a higher rate of people living in urban areas than India and Myanmar. Access to improved drinking water sources was the highest in Thailand and India and access to improved sanitation in Thailand and Myanmar (see Table 1).

**Table 1:** Socio-economic characteristics of the study countries.

Country	Gross National Income per Capita(2012) ^1^ (US$)	Children Aged <5 years Underweight for Age (2012) ^2 ^(%)	Mean Years of Schooling (2010) ^1^	Net primary School Enrolment Rate (2005–2011) ^2^ (%)		Living in Urban Areas (2011)^ 2^ (%)	Access to Improved Drinking Water Sources (2011) ^2 ^(%)	Access to Improved Sanitation (2011)^ 2 ^(%)
				Male	Female			
1. India	3285	43.5	4.4	99	98	31	92	35
2. Indonesia	4154	18.6	5.8	97	93	51	84	59
3. Myanmar	1817	22.6	3.9	--	--	33	84	77
4. Thailand	7722	7.0	6.6	90	89	34	96	93

Notes: **^1^** United Nations Development Program (UNDP) (2013) [27]; **^2^** World Health Statistics 2013 [28].

### 2.2. Measures

The GSHS questionnaire was used in this study. The questionnaire included modules on tobacco, alcohol and other drug use; dietary behaviors; hygiene; mental health; physical activity; sexual behaviours; unintentional injuries and violence; and protective factors and demographics [26].

#### *Oral and Hand Hygiene Behaviour* 

Oral and hand hygiene behaviour was assessed with four questions. The first question asked “During the past 30 days, how many times per day did you usually clean or brush your teeth?” (Response options were 1 = I did not clean or brush my teeth during the past 30 days, 2 = less than 1 time per day to 6 = 4 or more times a day). The remaining three questions asked “During the past 30 days, how often did you wash your hands before eating?” “…after using the toilet or latrine?” and “…how often did you use soap when washing your hands?” (Response options included 1 = never to 5 = always) [26].

#### *Eating Patterns* 

Hunger: A measure of hunger was taken from the question “During the past 30 days, how often did you go hungry because there was not enough food in your home?” (Response options were from 1 = never to 5 = always) [26].

Fruits: “During the past 30 days, how many times per day did you usually eat fruits, such as “country specific examples”?” (Response options were 1 = I did not eat fruits during the past 30 days, 2 = less than one time per day, 3 = 1 time per day to 7 = 5 or more times per day).

Vegetables: “During the past 30 days, how many times per day did you usually eat vegetables, such as “country specific examples”?” (Response options were 1 = I did not eat vegetables during the past 30 days, 2 = less than one time per day, 3 = 1 time per day to 7 = 5 or more times per day) [26]. Adolescents who reported that they consumed fruits (or vegetables) less than once a day were classified as having inadequate consumption patterns.

#### *Body Mass Index (BMI) Measurement* 

Height and body weight were assessed by self-reports. The international age-and gender specific child BMI cut-points were used to define overweight and obesity [29]. Respondents with BMI values corresponding to an adult BMI of ≥25.0 kg/m^2^ were classified as overweight [29].

#### *Substance Use Variables* 

Smoking cigarettes: During the past 30 days, on how many days did you smoke cigarettes? (Response options were from 1 = 0 days to 7 = all 30 days) [26]. Alcohol use: “During the past 30 days, on how many days did you have at least one drink containing alcohol?” (Response options were from 1 = zero days to 7 = all 30 days) [26]. Drugs: “During your life, how many times have you used drugs, such as glue, benzene, marijuana, cocaine, or mandrax?” (Response options were from 1 = zero time to 4 = ten or more times) [26].

#### *Physical Activity* 

Leisure time physical activity was assessed by asking: “Physical activity is any activity that increases your heart rate and makes you get out of breath some of the time. Physical activity can be done in sports, playing with friends, or walking to school. Some examples of physical activity are running, fast walking, biking, dancing, football. Do not include your physical education or gym class.”, “During the past 7 days, on how many days were you physically active for a total of at least 60 min per day?” and “During a typical or usual week, on how many days are you physically active for a total of at least 60 min per day? [26]”.

#### *Leisure Time Sedentary Behavior* 

This was assessed by asking participants about the time they spend mostly sitting when not in school or doing homework: “How much time do you spend during a typical or usual day sitting and watching television, playing computer games, talking with friends, or doing other sitting activities [26].” Sedentary behaviour was defined as three or more hours sitting in a day.

#### *Psychological Distress* 

Psychological distress was assessed with five items. Loneliness: “During the past 12 months, how often have you felt lonely?” (Response options have been from 1 = never to 5 = always). Suicide ideation: “During the past 12 months, did you ever seriously consider attempting suicide?” (Response option was 1 = yes and 2 = no). No close friends: “How many close friends do you have?” (Response options 1 = zero to 4 = three or more). Anxiety or worried: “During the past 12 months, how often have you been so worried about something that you could not sleep at night?” (Response options have been from 1 = never to 5 = always). Sadness: “During the past 12 months, did you ever feel so sad or hopeless almost every day for two weeks or more in a row that you stopped doing your usual activities?” (Response option 1 = yes and 2 = no) [26]. A psychological index was created by adding up all five items, and recoding the sum into low = no psychological distress, medium = 1 item of psychological distress and high = 2 or more psychological distresses endorsed.

#### *Protective Factors* 

Protective factors were assessed with five items on peer support at school, parental or guardian supervision, connectedness, and bonding. Peer support at school was assessed with the question: “During the past 30 days, how often were most of the students in your school kind and helpful?”. Parental or guardian supervision: “During the past 30 days, how often did your parents or guardians check to see if your homework was done?” Parental or guardian connectedness: “During the past 30 days, how often did your parents or guardians understand your problems or worries?” and Parental or guardian bonding: “During the past 30 days, how often did your parents or guardians really know what you were doing with your free time?” (Response options to these questions were from 1 = never to 5 = always) [26]. All five protective factor items were added up, and recoded into low, medium and high lack of protective factors.

### 2.3. Data Analysis

Each country sample was restricted to the age group 13 to 15 years, younger and older participants were excluded from the analyses. For each country, the overall response rates were 84%–93% (see Table 2).

**Table 2:** The response rate and age of participants, by country.

Country	Survey Year	Overall Response Rate ^1^	Age Groups in Years (%)			Mean Age of Final Sample
		N (%)	13 years	14 years	15 years	
1. India	2007	84	2,017 (29.9)	2,654 (38.4)	2,080 (31.7)	14.0
2. Indonesia	2008	93	1,072 (33.2)	1,253 (45.2)	542 (21.6)	13.9
3. Myanmar	2007	95	585 (37.1)	628 (34.3)	770 (28.6)	13.9
4. Thailand	2008	93	841 (37.1)	871 (36.2)	511 (26.7)	13.9

Note: ^**1**^ Overall response rate, the product of school and the student response rate, refers to the entire sample including those students outside the targeted age range of 13 to 15 years.

Data analysis was performed using STATA software version 11 (year of release 2009; Stata Corporation, College Station, TX, USA). This software has the advantage of indicating robust standard errors that account for the sampling design, i.e. cluster sampling owing to the sampling of school classes. The hygiene behaviour variables were recoded into two categories: (1) inadequate or sub-optimal hygiene behaviour (tooth brushing less than twice per day and never, sometimes or most of the time washing hands before meals, after toilet and with soap) and (2) optimal hygiene behaviour (tooth brushing more than once per day, always washing hands before meals, always washing hands after toilet, and always washing hands with soap). Associations between socio-demographic variables, health risk behaviours, psychological distress, and protective factors among school children were analysed calculating odds ratios (OR). Logistic regression was used for the analysis of the impact of explanatory variables for the four sub-optimal hygiene behaviour variables separately (binary dependent variables). All variables statistically significant at the *p* < 0.05 level in bivariate analyses were included in the multivariate model. In the analysis, weighted percentages are reported. The *p*-value of less than 5% is used to indicate statistical significance. Both the reported 95% confidence intervals and the p-value are adjusted for the multi-stage stratified cluster sample design of the study.

## 3. Results and Discussion

### 3.1. Sample Characteristics and Hygiene Behaviour

The total sample included 13,824 school children aged 13 to 15 years from four Southeast Asian countries. There were 51.2% male and 48.8% female school children. Overall, the proportions of school children reporting sub-optimal oral hygiene (<twice a day tooth brushing) (22.4%) was lower than the proportions reported for not always washing their hands regularly before meals (45.2%), after toileting (26.5%) and washing their hands with soap (59.8%). The proportions of sub-optimal oral hygiene (tooth brushing) varied across countries, with significantly higher rates of poor oral hygiene in India and Myanmar than in Indonesia and Thailand. Thai school children did not always wash their hands before eating (65.9%) and with soap (67.0%), which was more frequently than in any other country, while Indonesian school children were the poorest in washing hands after toilet (34.6%; see Table 3). Pearson correlation found that the strongest positive correlation was found between not always washing hands before meals and not always washing hands with soap (*r* = 0.35, *p* < 0.001), followed by not always washing hands with soap and not always washing hands after toilet (*r* = 0.22, *p* < 0.001), and not always washing hands before meals and not always washing hands after toilet (*r* = 19, *p* < 0.001). Correlations between poor oral hygiene (brushing teeth) and not always washing hands (before meals *r* = 0.10, *p* = 0.023; after toilet *r* = 0.06, *p* < 0.001; and with soap *r* = 0.14, *p* < 0.001) were generally lower.

**Table 3:** Demographic and hygiene behaviour of 13–15 years old participants, by country.

Country	Total (N)	Sex % ^1^		Brushed Teeth <twice a day ^1^	Wash Hands before Eating (not always) ^1^	Wash Hands after Toilet or Latrine Use (not always) ^1^	Wash Hands with Soap (not always) ^1^
		Male	Female				
1. India	6,751	57.9 (52.6–63.2)	42.1 (36.8–47.4)	45.2 (42.6–47.7)	33.0 (29.4–36.7)	15.0 (12.4–17.6)	57.0 (53.1–60.9)
2. Indonesia	2,867	49.5 (46.8–52.2)	50.5 (47.8–53.2)	11.3 (9.2–13.4)	46.9 (43.5–50.3)	34.6 (30.9–38.4)	64.0 (61.4–66.5)
3. Myanmar	1,983	50.0 (46.5–53.4)	50.0 (46.5–53.5)	27.6 (23.9–31.2)	18.6 (15.7–21.5)	20.4 (17.2–23.5)	38.2 (33.6–42.7)
4. Thailand	2,223	49.2 (44.3–54.0)	50.8 (46.0–55.7)	12.8 (10.4–15.1)	65.9 (58.5–73.3	26.7 (24.0–29.5)	67.0 (63.3–70.7)
Total	13,824	51.2 (49.1–53.2)	48.8 (46.8–50.9)	22.4 (20.9–24.0)	45.2 (42.6–47.9)	26.5 (24.4–28.5)	59.8 (57.7–61.8)

Note: **^1^** 95% CI.

**Table 4:** Bivariate and multivariate logistic regression of less than twice-a-day tooth brushing and not always washing hands before meals.

	All	Tooth brushing <2/day		Not always washing hands before meals	
	%	COR (CI 95%)	AOR (CI 95%)	COR (CI 95%)	AOR (CI 95%)
**Female**	48.8 (49.1–53.2)	1.00	1.00	1.00	1.00
**Male**	51.2 (49.1–53.2)	1.85 (1.66–2.07) ***	2.03 (1.71–2.41) ***	1.15 (1.03–1.28) *	1.04 (0.89–1.20)
**Age**					
13 years	34.2 (26.3–31.2)	1.00	1.00	1.00	
14	40.0 (37.4–41.8)	1.12 (0.99–1.26)	1.02 (0.89–1.35)	1.03 (0.91–1.18)	--
15 years	25.8 (23.2–28.4)	1.21 (1.07–1.36) **	1.03 (0.96–1.18)	1.09 (0.86–1.37)	--
**Went hungry**	4.4 (3.6–5.2)	1.16 (0.91–1.47)	--	1.12 (0.91–1.38)	--
**Substance use**					
—Current alcohol use	6.5 (5.4–7.6)	1.11 (0.82–1.50)	--	2.01 (1.54–2.63) ***	1.80 (1.25–2.20) **
—Current smoking	7.2 (5.9–8.4)	1.01 (0.77–1.32)	--	1.82(1.54–2.63) ***	1.21 (0.86–1.70)
—Ever used drugs	2.4 (1.9–2.9)	1.56 (1.08–2.44) *	1.05 (0.77–1.40)	1.82 (1.15–2.88) *	1.05 (0.50–2.41)
**Fruits less than once daily**	28.0 (26.2–29.9)	2.04 (1.77–2.35) ***	1.74 (1.41–2.13) ***	1.32 (1.15–1.50) ***	1.13 (0.95–1.34)
**Vegetables less than once daily**	14.0 (12.2–15.1)	1.97 (1.73–2.24) ***	2.11 (1.65–2.70) ***	1.56 (1.31–1.87) ***	1.21 (0.95–1.56)
**Physical activity**					
—<60 min daily (at 5 or more days a week)	81.5 (80.2–82.9)	1.08 (0.95–1.24)	--	1.52 (1.33–1.73) ***	1.26 (1.05–1.52) *
—Sedentary behaviour	30.3 (28.0–32.6)	0.89 (0.79–1.02)	--	1.79 (1.53–2.10) ***	1.75 (1.47–2.09) ***
**Overweight/obese**	8.2 (7.0–9.3)	1.04 (0.87–1.24)	--	1.42 (1.17–1.73) ***	1.35 (1.06–1.71) *
**Psychological distress**					
—Zero	70.6 (66.5–75.4)	1.00	1.00	1.00	1.00
—One	20.7 (19.3–22.1)	1.21 (1.07–1.38) **	1.01 (0.82–1.23)	1.05 (0.90–1.23)	0.94 (0.78–1.13)
—Two or more	8.7 (7.6–9.8)	1.39 (1.12–1.74) **	1.27 (0.95–1.70)	1.41 (1.12–1.77) **	1.02 (0.82–1.27)
**Lack of protective factors**					
—Low	41.1 (39.8–43.7)	1.00	1.00	1.00	1.00
—Medium	43.0 (41.8–44.1)	1.52 (1.33–1.72) ***	1.70 (1.39–2.08) ***	1.59 (1.41–1.79) ***	1.51 (1.28–1.78) ***
—High	15.9 (14.4–17.4)	2.13 (1.75–2.61) ***	2.26 (1.67–3.04) ***	2.39 (2.05–2.79) ***	2.39 (1.90–3.01) ***

Notes: *** *p* < 0.001; ** *p* < 0.01; * *p* < 0.05.

### 3.2. Sub-optimal Oral and Hand Hygiene Behaviour and Its Relationship with Social and Behavioural Variables

In multivariate analysis male gender (odds ratio: 2.03, 1.71–2.41), health risk behaviours (less than daily fruits and vegetable consumption: 1.74, 1.41–2.13 and 2.11, 1.65–2.70, respectively, and lack of protective factors (2.26, 1.67–3.04) were associated with sub-optimal tooth brushing. This shows a dose-response relationship with tooth brushing less than twice a day (1.70, 1.39–2.08); (2.26, 1.67–3.04). In terms of not always washing hands before meals, in multivariate analysis, health risk behaviour (alcohol use: 1.80, 1.25–2.20; inadequate physical activity: 1.26, 1.05–1.52, sedentary behaviour: 1.75, 1.47–2.09), being overweight or obese (1.35, 1.06–1.71) and lack of protective factors (2.39, 1.90–3.01) increased the risk of sub-optimal hand washing before meals (see Table 4). Further, in multivariate analysis the variable went hungry (or low socioeconomic status), health risk behaviours (smoking: 1.68, 1.27–2.24; fruits less than once daily: 1.24, 1.07–1.43), psychological distress (1.39, 1.06–1.82) and lack of protective factors (1.90, 1.57–2.29) were found to be associated with sub-optimal washing hands after toilet.

In terms of not always washing hands with soap, in multivariate analysis, health risk behaviours (smoking: 1.72, 1.36–2.19; fruits less than once daily: 1.38, 1.16–1.65; vegetables less than once daily: 1.26, 1.06–1.49 and inadequate physical activity: 1.30, 1.10–1.55) and lack of protective factors (2.11, 1.72–2.59) increased the risk of sub-optimal washing hands with soap (see Table 5).

**Table 5:** Bivariate and multivariate logistic regression of washing hands after toilet and washing hands with soap.

	Not always washing hands after toilet		Not always washing hands with soap	
	COR (CI 95%)	AOR (CI 95%)	COR (CI 95%)	AOR (CI 95%)
**Female**	1.00	1.00	1.00	1.00
**Male**	1.18 (1.01–1.38) *	1.05 (0.87–1.27)	1.16 (1.03–1.31) *	1.05 (0.89–1.23)
**Age**				
13 years	1.00		1.00	
14	1.02 (0.91–1.16)		0.98 (0.87–1.11)	
15 years	0.96 (0.80–1.14)	1.21 (1.07–1.36) **	0.98 (0.85–1.13)	--
**Went hungry**	1.74 (1.37–2.21) ***	1.63 (1.19–2.24) **	1.04 (0.77–1.42)	--
**Substance use**				
—Current alcohol use	1.38 (1.10–1.73) **	1.09 (0.79–1.27)	1.64 (1.24–2.16) ***	1.33 (0.93–1.88)
—Current smoking	2.25 (1.82–2.78) ***	1.68 (1.27–2.24) ***	1.77 (1.46–2.14) ***	1.72 (1.36–2.19) ***
—Ever used drugs	(1.43–2.95) ***	1.05 (0.54–2.14)	1.31 (0.84–2.06)	--
**Fruits less than once daily**	1.43 (1.27–1.62) ***	1.24 (1.07–1.43) **	1.65 (1.43–1.89) ***	1.38 (1.16–1.65) ***
**Vegetables less than once daily**	1.54 (1.30–1.83) ***	1.20 (0.99–1.46)	1.59 (1.37–1.83) ***	1.26 (1.06–1.49) **
**Physical activity**				
—<60 min daily (at 5 or more days a week)	1.53 (1.27–1.85) ***	1.27 (0.99–1.61)	1.39 (1.23–1.57) ***	1.30 (1.10–1.55) **
—Sedentary behaviour	1.13 (0.99–1.29)	--	1.21 (1.06–1.39) **	1.11 (0.93–1.32)
**Overweight/obese**	1.00 (0.79–1.27)	--	0.97 (0.81–1.17)	--
**Psychological distress**				
—Zero	1.00	1.00	1.00	
—One	1.43 (1.22–1.68) ***	1.29 (1.06–1.56) *	1.16 (0.95–1.41)	
—Two or more	1.63 (1.27–2.09) ***	1.39 (1.06–1.82) *	1.05 (0.83–1.32)	--
**Lack of protective factors**				
—Low	1.00	1.00	1.00	1.00
—Medium	1.42 (1.24–1.62) ***	1.34 (1.16–1.55) ***	1.58 (1.41–1.76) ***	1.57 (1.38–1.79) ***
—High	2.31 (1.97–2.70) ***	1.90 (1.57–2.29) ***	2.07 (1.74–2.46) ***	2.11 (1.72–2.59) ***

Notes: *** *p* < 0.001; ** *p* < 0.01; * *p* < 0.05.

## 4. Discussion

This study among in-school children in four Southeast Asian countries found a 22.4% prevalence of sub-optimal oral hygiene (<twice a day tooth brushing), which seemed similar to studies in other regions in nine African countries (22.7%) [9] and in some Pacific island states (22%–38%) [10]. The sub-optimal oral hygiene was significantly higher in India and Myanmar than in Indonesia and Thailand, which also is conform with previous local studies in India [3,7,8] and Thailand [6].

In terms of hand washing behaviour, the study found that the proportions reported for not always washing their hands regularly before meals was 45.2%, after toileting 26.5% and washing their hands with soap 59.8%. These findings compare somewhat with other regions, *i.e.*, in nine African countries 37.8% did not always wash their hands before eating, 41.6% did not always wash hands after toilet or latrine use and 65% did not always wash hands with soap [9] and in Pacific island states where 30% to 35% did not always engage in hand washing before eating [10]. Further, the study found that Thai school children more frequently did not always wash their hands before eating (65.9%) and with soap (67.0%) than in any other of the four countries, while Indonesian school children were the poorest in washing hands after toilet (34.6%). Indian school children were found to be practicing better hand hygiene behaviour (before eating and after toilet) but washing hands with soap was poor as found in some previous surveys among school children [22,23] and the general adult population in India [30]. Among the four study countries Myanmar was the best in hand washing before meals and washing hands with soap compared to the other three countries. A previous study has also highlighted significant increases in hand washing with soap and water after defecation from 1997 to 2001 in Myanmar, following the contribution of the “National Sanitation Week and Social Mobilisation for Sanitation and Hygiene” [31]. Poor hand washing behaviour was also found among elementary school children in Indonesia, 59.5% did not wash hands properly before eating and after visiting the toilet [32].

As found in previous studies [9,11,12], this study also found that males were at greater risk of sub-optimal tooth brushing than females. Contrary to a number of other studies [8,10,11,13,14,15,16], lower socioeconomic status (assessed here with hunger) was not found to be associated with sub-optimal tooth brushing. Further, health risk behaviours (less than daily fruits and vegetable consumption) as well as lack of protective factors were, as found in other studies [9,18,19,20], associated with sub-optimal oral hygiene. Regarding sub-optimal hand hygiene behaviour the study found an association with low socioeconomic status (or went hungry), as found in other studies [10,22]. In agreement with some other studies [9,10], this study found that health risk behaviours (substance use, inadequate fruits and/or vegetable consumption, inadequate physical activity, sedentary behaviour) as well as being overweight or obese were found to be associated with sub-optimal hand hygiene behaviour. In addition, psychological distress and lack of protective factors increased the risk of sub-optimal washing hands with soap, as also found in a few other studies [9,10].

This study found cross-national differences in the prevalence of sub-optimal hygiene behaviours. The observed variations could be related to the different public health programmes and cultural context in the study countries [10]. Further, the study found co-occurrence of oral and hand hygiene with general health risk behaviours suggesting that clustering of health behaviours may occur before adulthood [16]. The association between oral hygiene, general hygiene, SES, *etc*. may be explained by Dorri et al.’s model [24, p.266], namely, “socio-demographic factors, sex and education influence hygiene behaviours in adolescents through their impact on Sense of Coherence and peer social networks. In order to control the prevalence of common infectious diseases in Southeast Asian countries, the promotion of hand-washing with soap and tooth brushing with tooth paste should be emphasized. Interventions to facilitate health-related behaviours and an increase in protective factors should be geared to the most important risk factors or mediators of these behaviours.

## 5. Study Limitations

This study had several limitations. Firstly, the GSHS only enrolls adolescents who are in school. School-going adolescents may not be representative of all adolescents in a country as the occurrence of sub-optimal oral and hand hygiene behaviour may differ between the two groups. As the questionnaire was self-completed, it is possible that some study participants may have miss reported either intentionally or inadvertently on any of the questions asked, as over reported hygiene behaviour has been found in other studies [33]. This effect may have been reduced by the fact that study participants completed the questionnaires anonymously. Further, the assessment of risk factors of hygiene behaviour was limited and other factors could have been included [32,34,35,36]. Finally, since the data were collected in a cross-sectional survey we cannot, therefore, ascribe causality to any of the associated factors in the study.

## 6. Conclusions

The cross-national data on oral and hand hygiene behaviour from four Southeast Asian countries found sub-optimal hygiene behaviour (tooth brushing, hand washing before meals, after toileting and washing their hands with soap). Several risk factors of sub-optimal hygiene behaviour were identified such as low socioeconomic status, health risk behaviours, psychological stress, and lack of protective factors that can inform programmes in order to improve hygiene behaviour of this adolescent population. 
